# Physicochemical properties, cytotoxicity and bioactivity of a ready-to-use bioceramic repair material

**DOI:** 10.1590/0103-6440202304974

**Published:** 2023-03-06

**Authors:** Lívia Bueno Campi, Elisandra Márcia Rodrigues, Fernanda Ferrari Esteves Torres, José Maurício dos Santos Nunes Reis, Juliane Maria Guerreiro-Tanomaru, Mário Tanomaru-Filho

**Affiliations:** 1 Department of Restorative Dentistry, Araraquara Dental School, UNESP - São Paulo State University, Araraquara, SP, Brazil; 2 Department of Dental Materials and Prosthesis, Araraquara Dental School, UNESP - São Paulo State University, Araraquara, SP, Brazil

**Keywords:** Bioactivity, calcium silicate, cytotoxicity, physicochemical properties, X-ray microtomography

## Abstract

The aim of this study was to evaluate the physicochemical properties, cytotoxicity and bioactivity of a ready-to-use bioceramic material, Bio-C Repair (Angelus), in comparison with White MTA (Angelus) and Biodentine (Septodont). The physicochemical properties of setting time, radiopacity, pH, solubility, dimensional and volumetric changes were evaluated. Biocompatibility and bioactivity were assessed in Saos-2 osteoblast cell cultures by the MTT assay 3-(4,5-Dimethylthiazol-2-yl)-2,5-diphenyltetrazolium bromide), Neutral Red (NR), Alizarin Red (ARS), and cell migration tests. Statistical analysis was performed by ANOVA, Tukey or Bonferroni tests (α = 0.05). Bio-C Repair had the longest setting time (p < 0.05), but radiopacity and solubility were accordance with the ISO 6876/2012 standards, besides linear expansion. Bio-C Repair and MTA had similar volumetric change (p > 0.05); lower than Biodentine (p < 0.05). All the materials evaluated had an alkaline pH. Bio-C Repair was cytocompatible and promoted mineralized nodule deposition in 21 days and cell migration in 3 days. In conclusion, Bio-C Repair had adequate radiopacity above 3mm Al, solubility less than 3%, dimensional expansion, and low volumetric change. In addition, Bio-C Repair promoted an alkaline pH and presented bioactivity and biocompatibility similar to MTA and Biodentine, showing potential for use as a repair material.

## Introduction

Mineral Trioxide Aggregate (MTA) is mainly composed of tricalcium silicate, dicalcium silicate, and a radiopacifying agent ^1^. New calcium silicate-based materials are proposed to improve some properties such as consistency, dental discoloration, and long setting time ^1^. Biodentine (Septodont, Saint Maur des Fosses, France) is a commercial tricalcium silicate-based repair cement with a powder/liquid composition. The powder is composed of tricalcium silicate, dicalcium silicate, and zirconium oxide as a radiopacifier_._ Calcium chloride and a plasticizer were added in the liquid to decrease the setting time and improve its handling properties when compared to MTA ^2^. Biodentine allows hydroxyapatite deposition on its surface ^2^, but has high solubility and lower radiopacity than MTA ^3^ ^,^ ^4^.

Bio-C Repair (Angelus, Londrina, PR, Brazil) is a ready-to-use bioceramic material composed of tricalcium silicate, calcium aluminate, calcium oxide, zirconium oxide, iron oxide, silicon dioxide, and dispersing agent. Bio-C Repair is a pre-mixed cement ^5^, and the setting reaction occurs with moisture from the dentin and adjacent tissues ^6^. Bio-C Repair has cytocompatibility similar to that of tricalcium silicate-based materials ^7^, induces biomineralization, and shows appropriate clinical results ^8^. However, there are no studies evaluating the physicochemical properties of this material.

Therefore, the aim of this study was to evaluate the physicochemical properties, cytotoxicity and bioactivity of the ready-to-use bioceramic repair cement Bio-C Repair, in comparison with White MTA (Angelus, Londrina, PR, Brazil) and Biodentine. The proposed methodologies must contribute to the adequate clinical application of the repair material. The null hypothesis is that there is no difference in the physical-chemical and biological properties of the evaluated materials.

## Material and methods

The materials evaluated, their respective manufacturers and proportions used are described in Box 1.

**Box 1:**
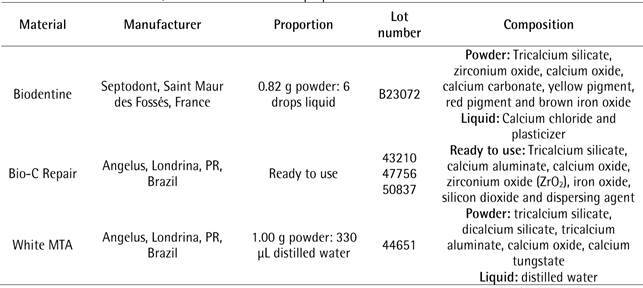
Endodontic materials, their manufacturers and proportions used

### Physicochemical properties evaluation

### Setting time

For each material, samples (n = 6) were fabricated in plaster molds type IV micro-granulated (Dentsply Indústria e Comércio Ltda, Petrópolis, Rio de Janeiro, Brazil) measuring 10 mm in diameter and 1 mm high. The plaster molds were immersed in distilled water for 24 hours before the test. A Gilmore needle weighing 100 ± 0.5 g and a diamond tip diameter of 2 ± 0.1 mm was used to determine the setting time in accordance with the ISO 6876:2012 standard. The setting time was calculated in minutes, from the final time of manipulation until the needle no longer marked the material surface. During the tests, the molds were kept in an oven at 37 °C and 95% humidity.

### Radiopacity

Samples of each material were fabricated (n = 6) with an internal diameter of 10 mm and height of 1 mm, and kept in an oven (37 °C, 95% humidity) for 48 hours. After this, they were placed on an occlusal film (Insight - Kodak Comp, Rochester, NY USA) together with an aluminum scale to be radiographed (X-ray Appliance GE 1000 - General Electric, Milwaukee, WI USA) at 60 kV, 7 mA, 0.32 pulses per second, and a focus-film distance of 33 cm. After processing and digitalizing the films, the samples were evaluated by using the UTHSCSA ImageTool for Windows version 3.00 software, to determine the radiopacity equivalence of the cements, in millimeters of aluminum (mm Al).

### Dimensional change

Cylindrical specimens of each material (n = 8) measuring 3.58 mm high by 3.00 mm in diameter ^9^ were fabricated and transferred to an oven (95% humidity and 37 ºC). After setting, the samples were removed from the molds and their height length was measured with a digital pachymeter (Mitutoyo). Afterwards, the samples were kept immersed in flasks containing 2.24 mL of distilled and deionized water (37 ºC) for 30 days (one sample per flask). After this period, the excess water was removed using absorbent paper, the samples were measured again and their final lengths were determined.

### Solubility

Based on Carvalho-Junior (2017), circular samples of the materials (n = 6) were fabricated, measuring 1.5 mm high and 7.75 mm in internal diameter ^9^. Nylon threads were inserted into the fresh material. The samples were kept in an oven (37 ºC and 95% humidity) for 48 hours. To obtain the initial mass, the samples were weighed on a precision balance (Ohaus Adventurer, Model AR2140, São Bernardo do Campo, SP, Brazil) until the mass had stabilized. After this, the samples were suspended by the nylon thread, in flasks containing 7.5 mL (one sample per flask) of distilled and deionized water ^3^ ^,^ ^10^ ^,^ ^11^, and kept in an oven (37 °C) for seven days. To obtain the final mass, the samples were removed from the distilled water, placed in a desiccator, and weight every 24 hours until the final mass had stabilized. The loss of mass was expressed in percent of original mass.

### pH

Polyethylene tubes measuring 10 mm high by 1 mm diameter were filled with the materials (n = 10), immersed in 10 mL of distilled and deionized water and kept in an oven for the experimental time intervals of 1, 7, 14 and 21 days. After each time interval, the specimens were removed from the flasks and put into new flasks each containing distilled water. The pH of the solution was measured using a calibrated digital pHmeter (Digimed, SP, Brazil). Flasks containing only distilled and deionized water were used as control.

### Volumetric change by micro-CT

The volumetric change test was based on a previous study^10^. Six specimens of each material (7.75 mm x 1.5 mm) were fabricated and kept in an oven (37ºC and 95% humidity) for 48 hours. After setting, the specimens were scanned by micro-CT (SkyScan 1176; Bruker-MicroCT, Kontich, Belgium). Thus, the samples were immersed in distilled water for the time intervals of 7 and 30 days, and new scanning were performed after each period. The scanning parameters used were: voltage of 80 kV, current of 300 μA, 18 μm pixel size, copper and aluminum (Cu + Al) filter, and rotation of 360°. The images obtained were reconstructed after determining the correction parameters for each material by using NRecon software (V1.6.4,7; Bruker, Belgium). So, the 3D images were superimposed on the different periods by using the Dataviewer software (V1.5.2.4; Bruker, Belgium), and quantitatively evaluated with the CTAn software (V1.11.8; Bruker, Belgium) regarding their volumetric change.

### Biological properties evaluation

*Cell culture and preparation of extracts*

For performing the MTT, Neutral Red and Alizarin Red assays, immortalized Saos-2 cells (osteoblast-like cells derived from human osteosarcoma) were used. Saos-2 (ATCC HTB-85) cells were cultured in T-75 flasks (Jet Biofil, Elgin, IL, USA) containing D-MEM medium (Sigma-Aldrich, St. Louis, MO, USA), supplemented with 10% of fetal bovine serum (FBS, Gibco, Life Technologies, Grand Island, NY, USA), penicillin (100 IU mL^-1^), streptomycin (100 lgmL^-1^) in 95% humidified atmosphere, 5% CO^2^ and 37°C until confluent. For the osteoinductive assays, the same medium was used supplemented with 50 lgmL^-1^ L-ascorbic acid (Sigma-Aldrich) and 10 mmol L^-1^ b-glycerophosphate (Sigma-Aldrich). Adherent cells at the logarithmic growth phase were detached by trypsin/ethylenediaminetetraacetic acid mixture (0.25%) (Gibco)at 37 °C for 3 min.

For cell viability analysis, 0.5 g of each material was measured on a precision balance, manipulated in the due proportions, and placed in the bottoms of wells of 12-well plates, which were stored in an oven (37 ˚C) until the materials had set completely. After this period, to prevent contamination, the plates were exposed to UV light for 30 minutes. Subsequently, 5mL of Dulbecco's Modified Eagle's Medium (DMEM - Sigma- Aldrich; St. Louis, Missouri, USA) was added to each well of the plates, and kept in an oven (37 ˚C, 95% humidity and 5% CO_2_) for 24 hours, for formation of the eluate of each material according to the ISO 10993-12:2012. After 24 hours, this medium was collected to obtain the dilutions of 1:1, 1:5, 1:10 e 1:15.

### (MTT assay) 3-4,5-Dimethylthiazol-2-yl) -2,5 -diphenyltetrazolium bromide)

Saos-2 cells were plated in the dilution of 1x10^5^ cells/mL in 96-well plates (TPP). Cells are exposed to the cements extracts or culture medium + 20% dimethyl sulfoxide (DMSO)-as positive control and DMEM serum-free medium as negative control. After 24h the medium was changed to DMEM + 5 mg/mL of MTT and the plates were left to incubate for a time interval of 3 hours. After this, the wells were washed with 1 mL phosphate buffer solution (PBS 1X) and to solubilize the formazan, 500 µl isopropyl alcohol (HCl: isopropyl alcohol, 0.04N) was added. An automatic miniplate reader (ELx800, Bio-Tek Instruments, Winooski, VT, USA) was used to measure the optical density at 570 nm. The experiment was repeated three times and performed in sextuplicate for each experimental group and outcome (n = 3/group).

### Neutral Red (NR)

Saos-2 cells (1x10^5^ cells/mL) were plated in 96-well plates (TPP) in D-MEM medium supplemented with 10% FBS. After the cells had remained in contact with the cement extracts in their different concentrations for 24 hours or culture medium + 20% DMSO (positive control) and DMEM serum-free medium (negative control), the extracts were replaced by 0.1 mL incomplete D-MEM medium (without FBS), containing 50µg NR/mL (Sigma-Aldrich). The plates were incubated at 37º C, 95% humidity and 5% CO_2_ for 3 hours. After this, the dye was removed and the colorimetric product was solubilized in 100 µL of 50% ethanol and 1% acetic acid solution (Sigma-Aldrich). The optical density was measured in the plate reader at 570 nm (Asys-UVM 340, Biochrom - MikroWin 2000, USA). The experiment was repeated three times and performed in sextuplicate for each experimental group and outcome (n = 3/group).

### Alizarin Red (ARS)

Saos-2 cells were plated (1x10^4^ cells/mL) in 24-well culture plates in DMEM medium supplemented with 10% FBS. Eluates of the materials were prepared and inserted into osteogenic DMEM culture medium (DMEM 10% FBS; 100 IU/mL penicillin; 100 mg/mL streptomycin; 0.023 g/mL β-Glycerophosphate; 0.055 mg/mL ascorbic acid - Sigma Chemicals St Louis MO, USA) for 21 days, renewed every 2 days. After the experimental time interval, the medium was aspired, the wells were washed with PBS 1X, and the cells were fixed in 70% ethanol at 4 ºC for 1 hour. The monolayers were washed twice with distilled water, and 0.3 mL of 40 mM Alizarin Red S (ARS, 2%- pH 4.1) was added. The plates were kept incubated at ambient temperature for 2 minutes. The dye was removed and the wells were carefully washed 4 times with 1 mL distilled water/well for 5 minutes. For quantitative analysis, the nodules were solubilized in 0.5 mL Cetylpyridinium chloride (Sigma-Aldrich) under agitation for 15 minutes. After homogenization, three aliquots of 100 µL from each well were transferred to a 96-well plate. Mineralized nodule formation was analyzed in a plate reader (ELx800, Bio-Tek Instruments), according to the absorbance determined at 562 nm. DMEM 10% SFB; β-glycerolphosphate; 0.055 mg/mL ascorbic acid as used as osteogenic control and DMEM medium with 10% fetal bovine serum as control group. Identical triplicates were prepared for each reaction, and the experiment was repeated three times independently (n = 3/group).

### Cell Migration

The Scratch Wound-Healing assay was performed to evaluate cell migration after exposure to the different restorative materials^12^. The cells were cultivated in 12-well plates containing α-MEM medium, 6x10^5^ cells/well. The culture plates were kept in an oven at 37 ºC, 95% humidity and 5% CO_2_ for 24 h until confluence was reached (24 hours). After this, the cells were removed with the use of a 200 µL tip (Universal Fit Pipette Tips, Corning Inc.), washed twice with PBS, and then exposed to the different restorative material extracts (0.2 µg/mL). The wells were photographed in the initial period and at time intervals of 1, 2 and 3 days using a microscope (ZeissAxiovert 100, 10X objective, Cambridge, UK). The images were analyzed with ImageJ software (National Institutes of Health, NIH, Bethesda, Maryland, USA) to determine the area of cell growth. The experiments were performed in quadruplicate and repeated at two different times (n = 4/group). Eight different fields per well were photographed and analyzed.

### Statistical analysis

The physicochemical properties data were submitted to the Shapiro-Wilk normality test and biological properties data were submitted to the Kolmogorov-Smirnov test. All the data were analysed with the GraphPad Prism statistical software package (GraphPad Software Inc.; San Diego, CA, USA). The physicochemical properties and ARS were submitted to one-way ANOVA and Tukey tests. MTT, NR and cellular migration were evaluated by two-way ANOVA and Bonferroni tests (α = 0.05).

## Results

Bio-C Repair had the longest setting time and the highest dimensional expansion (p < 0.05). Bio-C Repair had radiopacity higher than 3 mm Al and solubility lower than 3%, as recommended by the ISO 6876:2012. Bio-C Repair had higher radiopacity than Biodentine (p < 0.05) and the lowest solubility (p < 0.05). The volumetric change of Bio-C Repair were similar to MTA in both periods, which had lower values than Biodentine (p < 0.05) (Table 1). All the cements had an alkaline pH (Table 2).

**Table 1:** Setting time, radiopacity, solubility, dimensional change, and volumetric change values observed in endodontic materials (mean and standard deviation)

Materials/tests	Biodentine	Bio-C Repair	White MTA
Setting time (minutes)	35.14 (3.53)^b^	79.80 (0.84)^a^	25.17 (2.40)^c^
Radiopacity (mm Al)	2.30 (0.17)^c^	4.06 (0.19)^b^	4.80 (0.39)^a^
Solubility (% mass loss)	5.27 (0.37)^a^	2.92 (0.54)^c^	4.04 (0.77)^b^
Dimensional change (mm)	0.31 (0.23)^b^	1.38 (0.31)^a^	0.52 (0.14)^b^
Volumetric change (%) - 7 days	-4.18 (0.69)^a^	-0.29 (0.26)^b^	-0.41 (0.19)^b^
Volumetric change (%) - 30 days	-4.93 (0.74)^a^	-0.81 (0.38)^b^	-0.64 (0.21)^b^

Different letters on the same row represent significant differences between the different cements (p < 0.05)

**Table 2:** pH values observed in endodontic materials after storage in distilled and deionized water (mean and standard deviation)

Materials/ experimental periods	Biodentine	Bio-C Repair	White MTA	Control
1 day	11.63 (0.24)^a^	10.32 (0.16)^c^	11.09 (0.32)^b^	6.97 (0.29)^d^
7 days	10.46 (1.05)^b^	10.59 (0.17)^a^	10.20 (1.50)^c^	6.61 (0.51)^d^
14 days	10.00 (1.24)^a^	10.42 (0.08)^a^	9.40 (0.99)^b^	6.69 (0.41)^c^
21 days	10.03 (1.55)^ab^	9.28 (0.23)^b^	10.12 (0.61)^a^	6.27 (0.15)^c^
28 days	9.64 (1.34)^a^	9.73 (0.20)^a^	9.14 (0.66)^a^	6.76 (0.36)^b^

Different letters on the same row represent significant differences between the different cements (p < 0.05)

**Figure 1:**
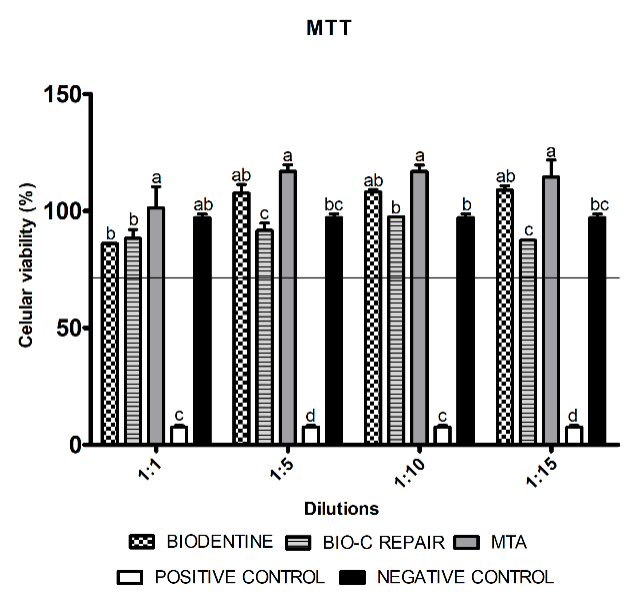
3-(4,5-dimethyl-thiazoyl)-2,5-diphenyl-tetrazolium assay (MTT). Cell viability (%) assessed by MTT test after 24 hours of exposure in different dilutions (1: 1, 1: 5, 1:10 and 1:15) of the cements and culture medium (negative control) in Saos-2 cells. Bars with different letters represent significant differences between groups in each concentration of the eluted material. The black line indicates the minimum level (70%) of the cell viability required to recognize the biomaterial as non-cytotoxic. Biodentine, Bio-C Repair, MTA - Mineral trioxide aggregate, Positive control: culture medium + DMSO 20%, Negative control: DMEM serum-free medium.

**Figure 2:**
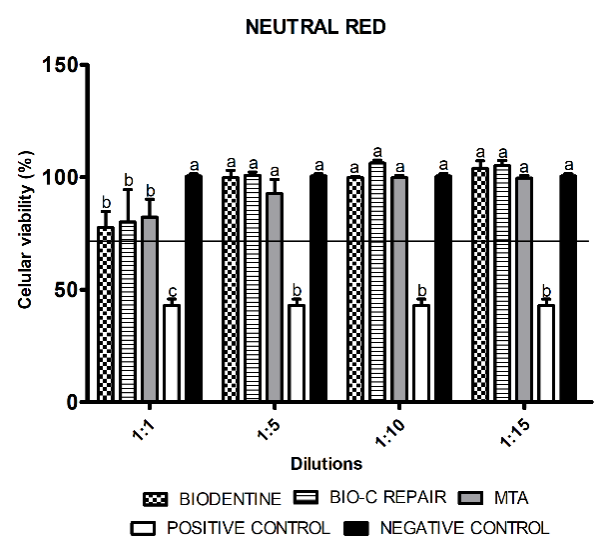
Neutral Red (NR). Cell viability (%) assessed by NR assay after 24 hours of exposure at different dilutions (1: 1, 1: 5, 1:10 and 1:15) of the cements. Bars with different letters represent significant differences between groups in each concentration of the eluted material. The black line indicates the minimum level (70%) of the cell viability required to recognize the biomaterial as non-cytotoxic. Biodentine, Bio-C Repair, MTA - Mineral trioxide aggregate, Positive control: culture medium + DMSO 20%, Negative control: DMEM serum-free medium.

**Figure 3:**
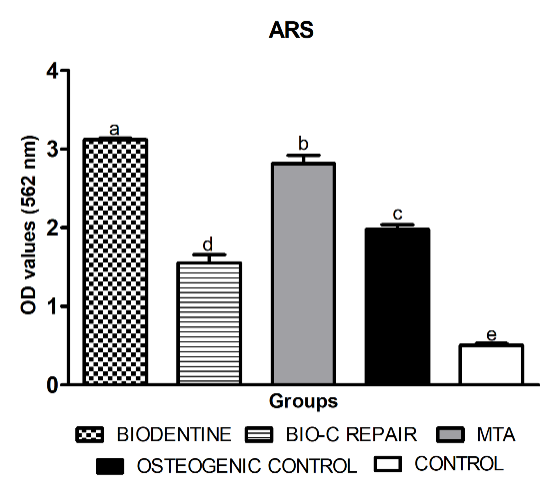
Graph of the Alizarin red color (ARS) statistical analysis after 21 days of osteogenic culture medium exposure to 1:10 dilutions of materials. Bars with different letters indicate a statistically significant difference between groups. Biodentine, Bio-C Repair, MTA - Mineral trioxide aggregate, Osteogenic control (DMEM 10% SFB; β-glycerolphosphate; 0.055 mg / mL ascorbic acid), control (DMEM medium with 10% fetal bovine serum).

**Figure 4:**
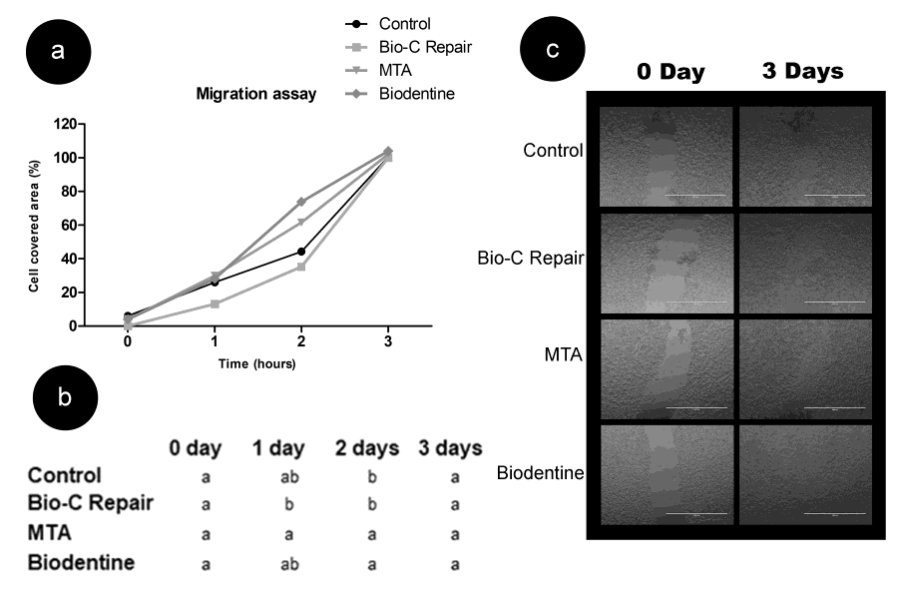
Cell migration. (a) Cell migration (as a percentage of cell coverage area) in Saos-2 cells after exposure to the 1:10 dilution of tested cements for different experimental periods (1, 2 and 3 days). (b) Statistical comparison of results: different letters in the columns indicate significant differences between the repair materials. (c) Representative images of cell migration at 0 and 3 days. Bar = 1000 μm.

In the dilution of 1:1, MTA, Biodentine and Bio-C Repair were similar to the negative control in the MTT assay (p > 0.05) (Figure 1). In the other dilutions, the cements showed higher (p < 0.05) or similar viability (p > 0.05) when compared to negative control. In the NR (1:1 dilution) all cements showed significantly lower cell viability than the control (p < 0.05). In other dilutions, the cytotoxicity was similar to the control (p > 0.05) (Figure 2). At 1:10 dilution (p < 0.05) all cements showed similar or higher viability to the negative control and for this reason, the 1:10 dilution was used for the ARS and cell migration. All the materials were capable of producing mineralized nodules, with higher values for Biodentine (Figure 3). MTA and Biodentine had larger cell migration in 2 days when compared with the control group. All the materials showed complete migration in 3 days (Figure 4).

### Discussion

In the present study, Bio-C Repair had some different physicochemical and biological properties than Biodentine and MTA. Therefore, our null hypothesis was rejected. The hydration of Bio-C Repair depends on the contact with the humidity ^2^. So, for the in vitro evaluation of setting time, the cement samples were inserted in plaster molds that were previously immersed in water, in accordance with ISO 6876:2012. Although Bio-C Repair had the longest setting time, our results showed values lower than the 120 minutes informed by the manufacturer. This is the first study evaluating the setting time of Bio-C Repair, with no parameters for comparison. However, previous studies have shown high setting time values for ready-to-use calcium silicate materials ^5^ ^,^ ^13^. One explanation for that could be the large quantity of water inside the pores of the plaster molds ^5^. The diffusion of water in these molds may vary according to the period and samples, differing from the amount of dentin fluids ^14^.

Biodentine had a radiopacity lower than 3 mm Al, as previously reported ^3^. Biodentine has 5% of zirconium oxide (ZrO_2)_ in its formula, which is insufficient to promote adequate radiopacity ^3^. The other cements evaluated had radiopacity higher than 3 mm Al. Bio-C Repair also has ZrO_2_ as radiopacifier. ZrO_2_ is inert in the hydration process, and has no influence on the physicochemical properties of materials ^2^, and its incorporation from 10% allows radiopacity higher than 3 mm Al ^15^.

Solubility and dimensional change in the materials may result in microleakage ^16^. Dimensional changes may be related to the expansion or shrinkage of materials after immersion in distilled water ^16^. Bio-C Repair had solubility below 3%, in accordance with ISO 6876 standard. Although there are no studies of the solubility of Bio-C Repair, Torres et al. observed volumetric loss for Bio-C Repair, which may be related to the solubility ^17^. Biodentine had solubility higher than 3%, which may be related to the presence of water-soluble polymer in its composition, which acts as a dispersant of cement particles, as previously demonstrated ^4^. Carvalho-Junior et al. ^17^ proposed, based on standard n. 57 of ANSI/ADA, reduction of the dimensions of the samples for the test of solubility using two circular samples of the cement immersed in 7.5 mL of distilled and deionized water. We have considered that the present study is based on Carvalho-Junior ^17^, but a sample of each cement immersed in 7.5 mL of water was used.

All the evaluated materials showed linear expansion. Three-dimensional evaluation of materials was performed by micro-CT, in order to complement the information obtained by conventional solubility and dimensional change tests ^10^.

Methodologies using micro-CT as a complementary test of the solubility and dimensional change of endodontic materials are previously proposed ^4^ ^,^ ^10^ ^,^ ^17^ ^,^ ^18^. Although there is no standardization for the volumetric change of materials and considering that the dimensional change should not exceed 1% according to ISO 6876, Bio-C Repair and MTA showed a low volumetric loss, with values below 1%. Biodentine showed a more significant volume reduction (above 4%). The higher volumetric loss observed to Biodentine may be related to its high solubility. The solubility and volumetric loss of Biodentine may also be associated with its high level of calcium and hydroxyl ion release after immersion ^4^. The low solubility and volumetric loss in addition to the dimensional expansion demonstrated by Bio-C Repair may be related to its hydration, water sorption and particle size ^1^. Furthermore, Bio-C Repair is a ready-to-use cement, which favor the homogeneity of the mixture, producing less porosity ^13^. This low porosity may lead to a low solubility, since these properties are associated ^18^.

The hydroxyl and calcium ion release makes the pH of the medium alkaline, and contribute to the antibacterial activity and osteogenic potential ^19^. In the current study, all the cements evaluated had alkaline pH in all the time intervals, as observed in other studies ^3^.

Saos-2 cells (osteoblast-like cells derived from human osteosarcoma) are used to evaluate the bioactive potential of tricalcium silicate-based materials ^12^. Cell viability was evaluated by means of 3-(4,5-Dimethylthiazol-2-yl)-2,5-diphenyltetrazolium bromide (MTT) and neutral red (NR) assays. In the present study, the materials evaluated were in accordance with ISO 10993-5: 2009 maintaining in vitro viability above 70% of cells exposed to the biomaterial. In the dilution of 1:10, MTA and Biodentine had similar cell viability and Bio-C Repair maintained viability next to the control. This result corroborated a previous study ^20^ that observed similar cell viability between Biodentine and MTA in the dilution of 1:10. Another investigation^21^ observed that Bio-C Repair showed cell viability similar to the negative control. The lower cell viability of Bio-C Repair in comparison with MTA may be related to the difference in the presentation of the materials ^22^. Part of ready-to-use materials may remain unhydrated promoting more leaching in the culture medium ^23^.

Biodentine showed higher levels of mineral nodule deposition, followed by MTA. Another study ^24^ also observed the greater formation of mineralized nodules for Biodentine than for MTA. MTA demonstrates the ability to induce the formation of calcium nodules ^12^ ^,^ ^22^. Bio-C Repair demonstrates mineralization potential in the subcutaneous tissue of rats ^21^, corroborating the bioactivity observed in the present study.

Endodontic reparative materials must have the capacity to promote cell migration promoting repair ^25^. In the present study, complete cell migration occurred after 3 days of exposure to the extracts of Bio-C Repair, MTA and Biodentine. The complete migration of Saos-2 cells was observed when exposed to the extract of Biodentine ^12^. Bio-C Repair allowed a complete migration of mesenchymal cells after 3 days^21^, corroborating the present study.

Therefore, it was possible to conclude that Bio-C Repair had radiopacity above 3mm Al and solubility below 3% *according to ISO 6876/2012*, dimensional expansion, and low volumetric loss. In addition, Bio-C Repair was capable of promoting alkaline pH and showed bioactivity and biocompatibility similar to MTA and Biodentine, showing potential for use as reparative material.
